# Advances in smart stimuli-responsive materials for oral wound healing

**DOI:** 10.3389/fchem.2025.1725373

**Published:** 2025-12-05

**Authors:** Xinyun Liu, Xi Huang, Ningyi Liu, Luanxin Zhu, Shuya Zhao, Shijia Tang, Penglai Wang

**Affiliations:** 1 School of Stomatology, Xuzhou Medical University, Xuzhou, Jiangsu, China; 2 Department of Prosthodontics, The Affiliated Stomatological Hospital of Nanjing Medical University, State Key Laboratory Cultivation Base of Research, Prevention and Treatment for Oral Diseases, Jiangsu Province Engineering Research Center of Stomatological Translational Medicine, Nanjing, Jiangsu, China; 3 Department of Pediatric Dentistry, Nanjing Stomatological Hospital, Affiliated Hospital of Medical School, Nanjing University, Nanjing, China

**Keywords:** oral wound healing, stimuli-responsive dressings, wound microenvironment, intraoral signals, external triggers

## Abstract

Oral wounds, particularly those coupled with bone defects like osteoradionecrosis and periodontitis, present a profound clinical challenge. While conventional biomaterial dressings offer basic therapeutic benefits, their static nature hinders dynamic interaction with the complex wound microenvironment, where factors like fluctuating pH and enzymatic activity impair healing. This review focuses on the development of “smart” stimuli-responsive dressings that overcome this limitation. These advanced systems are engineered to sense specific intraoral signals, such as pH, reactive oxygen species, or enzymes, or external triggers like light, enabling on-demand drug release and active wound microenvironment reprogramming. We critically synthesize recent progress in their design, stimuli-responsive mechanisms and therapeutic application, with a dedicated emphasis on bone-related oral pathologies. Furthermore, the review addresses the critical translational challenges and future prospects for bridging material innovation with clinical needs, aiming to facilitate next-generation regenerative therapies for oral and craniofacial defects.

## 1 Introduction

The oral cavity, serving as the gateway to the human body and the starting point of the alimentary tract, plays a vital role in food intake and speech. As a non-enclosed space with a naturally bacterial-rich environment, it is consistently exposed to diverse mechanical stimuli. An epidemiological study of maxillofacial soft tissue injuries in Beijing public hospital emergency departments from 2017 to 2018 revealed that the highest incidence rate was among patients under 10 years old (44.2%). Among 5,949 patients, 2021 (34.0%) presented with concomitant dental trauma, while 31 (0.5%) had maxillofacial bone fractures (Guo et al., 2021). In fact, various conditions, such as trauma, post-tumor resection, ulcers, tooth extraction, and surgical procedures can lead to oral wounds and defects. These injuries undoubtedly impair patients’ normal daily functions and significantly reduce their quality of life. Current clinical management typically involves debridement, hemostasis, and suturing. However, these methods cannot fully isolate the wound from the oral bacterial environment, and large defects often cannot achieve primary healing (Farahani and Shafiee, 2021). Persistent non-healing wounds cause pain and discomfort, while maxillofacial injuries may lead to psychological distress, collectively reducing patients’ productivity and imposing a substantial economic burden on society.

**SCHEME 1:**
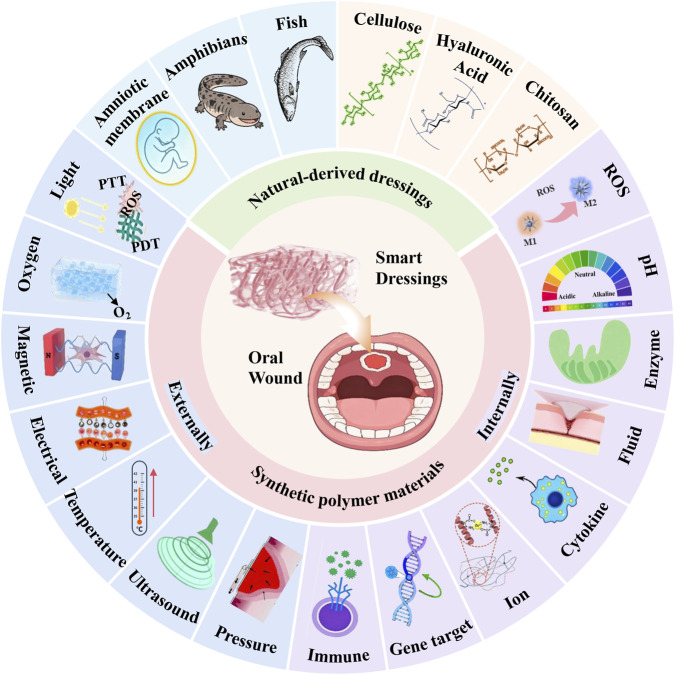
Schematic illustration of recent advances in smart stimuli-responsive dressings for oral wound healing.

Wound dressings have been used for an extensive period to cleanse, cover and protect wounds from external threats. They serve to cover the injured site and provide a temporary barrier against external infections, with the primary objectives of maintaining wound cleanliness, ensuring that the population of opportunistic pathogens remains within a safe range, and acting as an inductive template to guide the reorganization of poorly differentiated cells in the corresponding tissue, followed by the infiltration and integration of the target tissue to achieve optimal healing outcomes. Simultaneously, the dressing must be easy to replace, enabling practitioners to change it during practical operations without causing additional harm. In summary, an ideal dressing should possess the ability to provide adequate moisture, appropriate temperature, and a suitable pH environment, while also being easily removable (Liang et al., 2021). Substantial evidence indicates that the use of wound dressings can significantly reduce the incidence of infection and prevent scar contracture (Movaniya et al., 2021). Therefore, selecting appropriate wound dressings is essential for promoting rapid healing of intraoral wounds and preventing related complications. Hydrogels, films, and liquid dressings are among the commonly used types of moist wound dressings (Wang et al., 2020; Wang P. et al., 2024). However, the complexity of the intraoral wound microenvironment is characterized by several challenging features, including a naturally colonized bacterial environment, high levels of reactive oxygen species (ROS), low pH, abnormal matrix metalloproteinase activity, high humidity and considerable mobility of surrounding tissues (Shao et al., 2023). These factors collectively increase the risk of infection and can readily impede processes such as angiogenesis, ultimately leading to impaired wound healing. Currently available clinical wound dressings are often inadequate in addressing these specific intraoral conditions, resulting in suboptimal therapeutic outcomes or even the emergence of drug resistance. These limitations pose significant challenges for the clinical application of intraoral wound dressings, while also informing new design strategies for future development.

Ordinary “responsive materials” refers to simple, often continuous physical or chemical changes (e.g., a hydrogel swelling in moisture), while “smart stimuli-responsive” describes materials engineered to sense specific pathological cues (e.g., pH, enzymes) or external triggers and execute complex, on-demand functions, such as precise drug release or self-healing to actively reprogram the wound microenvironment and enhance regeneration. To address the limitations of current clinical intraoral wound dressings, researchers are actively developing smart stimuli-responsive wound dressings designed to adapt to the unique intraoral environment, characterized by its naturally colonized microbiota, high levels of ROS, low pH, MMP activity, high humidity, and dynamic movement of surrounding tissues, as well as external stimuli such as light, magnetic fields, electricity, temperature, and ultrasound. These intelligent dressings enable precise and on-demand drug release, thereby reducing the risk of antibiotic resistance associated with high-dose systemic administration and improving the compromised microenvironment of intraoral wounds.

This review aims to provide a clinically oriented overview of both conventional and smart responsive hydrogel dressings, highlighting their respective advantages and future prospect in the management of intraoral wounds. Specifically, it will examine the distinctive features of the intraoral wound milieu and the factors that impede healing, with a focused discussion on the application of smart responsive dressings in intraoral wound repair. Unlike broader reviews on wound dressings, our work specifically addresses the unique challenges of the oral microenvironment, a dynamic, wet and microbiologically complex milieu that is fundamentally different from the skin and critically evaluates how smart materials can be engineered to overcome these challenges (Scheme 1).

## 2 Characteristics of oral wounds and dressing design considerations

The oral mucosa and skin epithelium share similar structural organizations, yet they exhibit critical structural and functional differences. Unlike skin epithelium, which is composed of a keratinized epidermal layer, dermis, and subcutaneous tissue, the oral mucosa consists of stratified squamous epithelium, a basement membrane, the lamina propria, and a submucosal layer (Zhang Y. et al., 2025). With the exception of regions subjected to significant mechanical stress, such as the palate and gingiva, which are keratinized, most oral mucosal surfaces are non-keratinized. Consequently, while wound healing in both tissues follows similar phases, hemostasis, inflammation, proliferation, and remodeling, significant differences exist in their genomic profiles and healing kinetics. The distinct genomic expression patterns in oral mucosa contribute to its accelerated healing and minimal scar formation (Chen et al., 2010). Specifically, the oral mucosa demonstrates a reduced sensitivity to inflammatory stimuli, with lower levels of infiltration by macrophages, T lymphocytes, and neutrophils. Moreover, expression of transforming growth factor-beta 1 (TGF-β1) is relatively low in oral epithelial cells, which helps suppress the formation of hypertrophic scars commonly observed in cutaneous wounds (Turabelidze et al., 2014). Saliva, a unique weakly acidic (pH = 5.5–7) buffer in the oral cavity, contains a variety of bioactive components, including but not limited to antimicrobial peptides, pro-healing peptides, and growth factors, that facilitate wound healing by promoting fibroblast proliferation and migration, as well as enhancing keratinocyte turnover. This distinctive biochemical environment makes oral wounds a relatively ideal model for studying wound healing. However, the oral environment also presents significant challenges. Due to eating, drinking, and continuous salivary secretion, the oral cavity remains persistently moist and hosts a diverse microbiota, resulting in a constant exposure to microorganisms. In this open and non-sterile environment, wounds are highly susceptible to colonization by pathogenic bacteria. Excessive bacterial accumulation can lead to infection, impaired wound contraction, and scar formation. In more severe cases, systemic complications such as bacteremia, sepsis, or even endocarditis may occur. Furthermore, physiological movements such as those of the lips, cheeks, and tongue, along with mandatory masticatory activity from jaw motion, impose dynamic mechanical forces that can compromise the stability and functionality of wound dressings, affecting both their retention and therapeutic efficacy.

In response to the unique challenges of the oral environment, an ideal wound dressing for oral applications should meet the following requirements: (1) Excellent biocompatibility, (2) Effective sealing and barrier properties, (3) Appropriate mechanical properties, (4) Strong tissue adhesion, (5) High stability in moist conditions, (6) Ability to resist microbial penetration, (7) Promotion of wound healing, (8) Ease of removal without causing secondary trauma, and (9) Ease of clinical handling and application. Beyond these fundamental characteristics, smart responsive dressings offer unique advantages such as controllable drug release and adaptive functionality in response to environmental changes, further enhancing their therapeutic potential in the dynamic oral wound microenvironment.

## 3 Natural-derived dressings

### 3.1 Animal-derived dressings

#### 3.1.1 Amniotic membrane

Amniotic Membrane (AM), derived from the cytotrophoblast, constitutes the innermost layer of the fetal membrane. It is composed of a single layer of epithelial cells, a thick basement membrane, a stromal matrix rich in collagen, as well as dispersed fibroblasts and undifferentiated mesenchymal cells. Moreover, AM is endowed with an abundance of growth factors and collagen, which collectively promote epithelial cell proliferation (Hazarika et al., 2022; Odet et al., 2021). Its innate anti-inflammatory, antimicrobial, and anti-angiogenic properties make it an ideal biological material for burn and wound healing applications. Notably, the laminin structure of AM closely resembles that of native human tissues such as oral mucosa, which has garnered significant interest in its use for oral wound management (Mohan et al., 2017). To mitigate potential infection risks associated with traditional glycerol preservation, recent studies have proposed combining tissue preservation with gamma-irradiation sterilization. For instance, Naoya Arai et al. developed a super-dried AM processed using far-infrared and microwave treatment followed by gamma-ray sterilization, enabling long-term storage at room temperature. Alternatively, novel sterilizing agents such as acetic acid have also been explored for AM processing (Arai et al., 2012; Riau et al., 2010).

**FIGURE 1:**
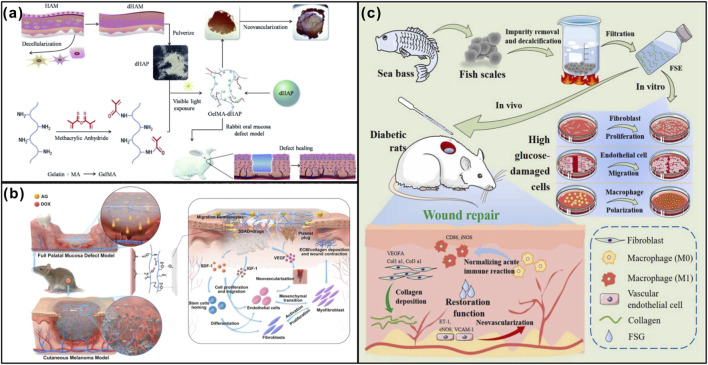
Animal-derived dressings promote wound healing. **(a)** Efficacy of GelMA–dHAP as a wound treatment on the rabbit oral mucosa defect model. Adapted from Ref. (Zhang et al., 2019), Copyright 2019, Royal Society of Chemistry. **(b)** Schematic diagram showing the possible mechanism by which the drug-loaded SSAD hydrogel promotes wound healing. Adapted from Ref. (Liu X. et al., 2022), Copyright 2022, Elsevier. **(c)** Schematic diagram of FSG prepared from sea bass scales promote wound healing. Adapted from Ref. (Wang H. et al., 2024), Copyright 2024, Elsevier.

Owing to the presence of anti-inflammatory proteins and antimicrobial peptides, AM exhibits notable antibacterial and anti-inflammatory effects, thereby facilitating wound healing. However, its clinical application in mucosal defect repair, particularly in the oral cavity, is limited by its thinness, mechanical fragility, difficult handling, and the complexity of the oral environment. To overcome these limitations, researchers have incorporated decellularized human amniotic membrane particles (dHAP) into methacrylated gelatin (GelMA) to fabricate a three-dimensional (3D) porous GelMA-dHAP composite scaffold. The adhesive properties of GelMA allowed the scaffold to effectively adhere to oral mucosal wounds, while the dHAP component enhanced rapid vascularization and promoted mucosal regeneration (Figure 1a) (Zhang et al., 2019).

#### 3.1.2 Amphibians

The Chinese giant salamander (*Andrias davidianus,* ADS) is a large, rare amphibian species endemic to China. Its skin secretes a substantial amount of bioactive substances that have been shown to promote wound healing (Yan et al., 2018). Yuqing Liu et al. reported on the wet-adhesion mechanism of a dried granular natural bioadhesive derived from the skin secretion of ADS, which contributes to its hemostatic effect. The dried ADS granules absorb blood, remove fluids and the surface liquid layer from the wound site, and form strong bonds to adhere firmly. Upon blood absorption, the ADS swells and physically blocks the wound to achieve hemostasis. The hydrophobic nature of the ADS hydrogel further prevents blood penetration. Additionally, the broadly distributed charges on ADS proteins help concentrate coagulation factors around the adhered hydrogel, facilitating clotting. ADS can also interact directly with cellular components in the blood to promote hemostasis (Liu et al., 2022a). Xiang Liu et al. developed a biomimetic adhesive hydrogel (SSAD) extracted from the skin secretions of the Chinese giant salamander, loaded with agents such as aminoguanidine or doxorubicin. SSAD contains a range of growth factors that promote cell migration and proliferation (Figure 1b) (Liu X. et al., 2022). Hui Yang et al. identified a novel antimicrobial peptide, AdCath, from the skin secretion genes of the Chinese giant salamander. Both recombinant and synthetic mature AdCath peptides were shown to disrupt bacterial cell membranes, demonstrating significant antimicrobial activity (Yang et al., 2017). In summary, skin secretions from the Chinese giant salamander show promising potential in wound healing therapies. Similarly, the Chinese giant lizard has also been studied for its bioactive skin secretions. Ximu Zhang et al. used cryogenic ball-milling to process the skin secretions of the Chinese giant lizard into a hemostatic powder, SSAD. This powder promotes wound healing by accelerating the aggregation of platelets and solid blood components, thereby achieving effective hemostasis (Zhang X. et al., 2022).

#### 3.1.3 Fish

Fish skin is rich in proteins and various trace elements, making fish skin grafts a promising biomaterial with enhanced wound healing capacity and inherent antimicrobial activity. As summarized by Kannan Kamala, bioactive compounds present in fish skin possess the ability to bind pro-inflammatory cytokines. Additionally, omega-3 fatty acids in the skin suppress inflammatory processes by modulating the expression of TGF-β1, thereby exerting both antibacterial and anti-inflammatory effects. Fish skin is also abundant in collagen; during its degradation, collagen breakdown releases fragments that stimulate fibroblast development, as well as factors that promote angiogenesis and re-epithelialization (Kamala et al., 2024). Beyond fish skin, Wang Haonan et al. extracted a natural composite hydrogel from fish scales, termed Fish Scale Gel (FSG). FSG influences macrophage polarization by modulating gene expression, thereby facilitating wound healing even under high-glucose conditions. Furthermore, FSG promotes the migration of endothelial cells and enhances the proliferation of fibroblasts, collectively accelerating the wound repair process (Figure 1c) (Wang H. et al., 2024).

### 3.2 Natural polymer-derived dressings

Natural polymers are derived from a wide range of sources and exhibit structural similarities to the extracellular matrix (ECM) (Wang et al., 2023). They can generally be classified into three major categories: polysaccharides, polypeptides, and proteins. These materials offer favorable biocompatibility and biodegradability, which can contribute to desirable bioactivity when used in intraoral dressings. However, their performance is highly variable due to inconsistencies between batches and sources, often leading to unpredictable clinical outcomes.

#### 3.2.1 Cellulose

As one of the most abundant polysaccharides in nature, cellulose is widely derived from both plant and bacterial sources. Bacterial cellulose (BC) is a natural polymer synthesized through static or dynamic fermentation by specific microorganisms (Wang X. et al., 2024). It exhibits notable hygroscopic properties, maintaining its structural and mechanical stability even upon reaching critical saturation hydration. BC demonstrates high hydrophilicity, permeability, and compressive strength, making it a more suitable candidate for wound dressing applications compared to plant-based cellulose. In terms of physical morphology, BC features a 3D nanofibrous network microstructure. When produced via static fermentation, it forms a macroscopic gel-like film. Chemically, BC consists of linear chains of glucose units linked by β-1,4-glycosidic bonds. Singh et al. have conducted extensive research on the application of BC in oral wound care. They initially developed an on-demand adhesive layered patch composed of BC and poly (amidoamine)-grafted-bisazide (PDz), which demonstrated prolonged adhesion to moist and soft mucosal surfaces. Subsequent improvements led to the fabrication of a fibrillated BC-PDz composite hydrogel, significantly broadening its applicability (Singh et al., 2021; Singh et al., 2023). However, these studies could not overcome the inherent lack of antimicrobial activity in BC. To address this limitation, Paul-Octavian et al. developed a biocomposite using poly (N-isopropylacrylamide)/poly (vinyl alcohol) nanoparticles supported on BC membrane and chitosan membrane. This system demonstrated considerable potential as an alternative wound dressing support, offering enhanced biocompatibility and antimicrobial functionality (Stanescu et al., 2021).

#### 3.2.2 Hyaluronic acid (HA)

**FIGURE 2:**
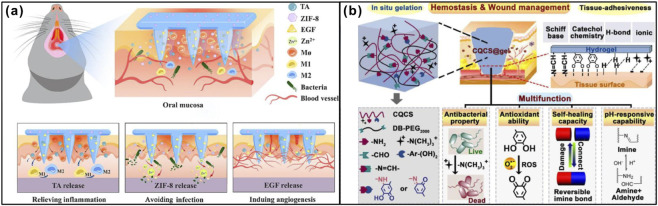
Natural polymer-derived dressings promote wound healing. **(a)** A multifunctional, soluble HA microneedle patch was prepared to promote oral ulcer healing. Adapted from Ref. (Ge et al., 2023), Copyright 2023, Elsevier. **(b)** Multifunctional CQCS@gel for hemostasis and wound management. Adapted from Ref. (Fang et al., 2023), Copyright 2023, Elsevier.

HA is a naturally occurring glycosaminoglycan and a major component of the ECM. It plays multiple critical roles in wound healing, including serving as a primary ligand for the CD44 receptor to mediate cell-cell interactions, promoting the expression of inflammatory cytokines, stimulating angiogenesis, and activating keratinocytes and fibroblasts to facilitate tissue repair (Kawano et al., 2021). In the field of oral medicine, HA is widely used in the treatment of mucosal diseases. For instance, Gengigel® gel, containing 0.2% HA, has been applied in patients with Behçet’s disease or recurrent aphthous ulcers, demonstrating significant clinical benefits such as reduced ulcer count, alleviated pain, and shortened healing time (Lee et al., 2008). Wenhui Ge et al. developed a multifunctional and soluble HA-based microneedle patch to promote the healing of oral ulcers. The tips of the microneedles were loaded with triamcinolone acetonide (TA) and epidermal growth factor (EGF) to suppress inflammation and stimulate angiogenesis, respectively. Additionally, zeolitic imidazolate framework-8 (ZIF-8) was incorporated into the patch’s base layer to enable the controlled release of Zn^2+^ for antibacterial effects. Experimental results confirmed the patch’s excellent anti-inflammatory, antimicrobial, and tissue regeneration capabilities (Figure 2a) (Ge et al., 2023). HA-based formulations have also been applied in post-tooth extraction wound management. Studies using 0.8% and 0.2% HA gels (Gengigel®) and 0.01% HA spray on extraction sockets consistently reported higher healing rates compared to control groups. Although the gel formulations appeared more effective, the difference in efficacy between the gel and spray was not statistically significant (Gocmen et al., 2015).

Despite its favorable biological activities, HA as a natural polymer has limitations such as poor mechanical strength, high swelling ratio, smooth surface properties, and susceptibility to enzymatic degradation, which necessitate further modification for optimized clinical performance.

#### 3.2.3 Chitosan (CS)

CS, typically derived from crustacean exoskeletons or fungal cell walls, is the only naturally occurring cationic polysaccharide with alkaline nature. It exhibits strong antimicrobial and hemostatic properties, along with excellent biodegradability and biocompatibility, making it widely applicable in the form of hydrogels, films, and other biomaterial constructs. CS promotes fibroblast proliferation and facilitates organized collagen deposition, effectively accelerating wound healing and reducing scar formation.

The therapeutic efficacy of chitosan in mucosal wound treatment has been extensively validated. Both chitosan-containing sprays (Ke et al., 2021) and circular chitosan patches (Kılıç et al., 2013) have demonstrated significant benefits in hemostasis, pain relief, and enhanced healing. Further studies have confirmed that chitosan application promotes the healing of oral mucosal ulcers (Luo et al., 2020; Zheng et al., 2020). When used as a dressing following tooth extraction, CS-based Hemcon® dressings resulted in improved healing outcomes not only in general patients but also in those undergoing antiplatelet therapy (Pippi et al., 2017). Wen Fang et al. developed an injectable hydrogel using catechol-functionalized quaternized chitosan and dibenzaldehyde-terminated polyethylene glycol (DB-PEG2000). This formulation exhibited strong tissue adhesion and physical sealing capabilities that enhanced hemostasis, in addition to promoting collagen deposition and angiogenesis, thereby significantly improving wound healing (Figure 2b) (Fang et al., 2023). Owing to its high adhesiveness and antimicrobial activity, chitosan is particularly suitable for the challenging intraoral environment. Numerous chitosan-based composites have been developed and applied in wound healing, yet broader utilization of CS materials in the field of oral wound dressings warrants further exploration. Although traditional natural dressings offer favorable biocompatibility and biodegradability, they face considerable limitations in the dynamic and complex oral environment, often failing to achieve stable and precise regulation of healing. Therefore, the functional design of materials, endowing them with environmental responsiveness, programmable drug release, and adaptive mechanical behavior, has emerged as a central direction in the development of next-generation oral dressings.

In this context, “smart stimuli-responsive dressings” have gained attention as an advanced research direction for promoting healing in complex oral wounds due to their ability to dynamically sense and respond to changes in the oral microenvironment. By incorporating specific functional groups or structures, these materials can sensitively react to common physiological or pathological stimuli in the oral cavity, such as pH fluctuations, enzymatic activity, temperature variations, microbial metabolites, or redox status, and adjust their physicochemical properties (e.g., swelling capacity, adhesiveness, degradation rate, and drug release kinetics) in real time. This enables spatiotemporally precise control over treatment strategies. Based on this “smart response” concept, a variety of synthetic polymer materials have been designed, offering intelligent solutions to accelerate oral wound healing.

## 4 Synthetic polymer materials

Smart responsiveness refers to the ability of biomaterials to dynamically and rapidly recognize and respond to changes in external stimuli or the internal pathological microenvironment (Ahmadi et al., 2020). In this section, we provide a concise overview of various stimuli-responsive strategies employed in synthetic polymer-based systems, which can be broadly categorized into: (1) Externally triggered response strategies, including light, temperature, magnetic fields, electricity, ultrasound, ozone and mechanical pressure; (2) Internally triggered response strategies: such as enzymes, ROS, pH, blood components, cytokines, ions, genetic signals and immune activity.

### 4.1 Externally triggered response strategies

By applying and controlling external stimuli such as light, magnetic fields, electrical stimulation, and ultrasound, wound dressings can be engineered to respond to the local environment by releasing active substances and transmitting therapeutic and/or pro-regenerative signals into the body. This enables precise regulation of different stages of wound healing, ultimately accelerating the recovery process.

#### 4.1.1 Light-responsive strategies

Light-responsive strategies leverage the photocatalytic activity and photothermal conversion properties of photosensitive materials incorporated into wound dressings. These materials enable photodynamic therapy (PDT) and photothermal therapy (PTT), both of which contribute to enhanced wound healing. Additionally, photobiomodulation (PBM) can promote tissue repair by inducing the expression of key growth factors and modulating the balance between pro-inflammatory and anti-inflammatory cytokines (Khan and Arany, 2015).

##### 4.1.1.1 PDT

**FIGURE 3:**
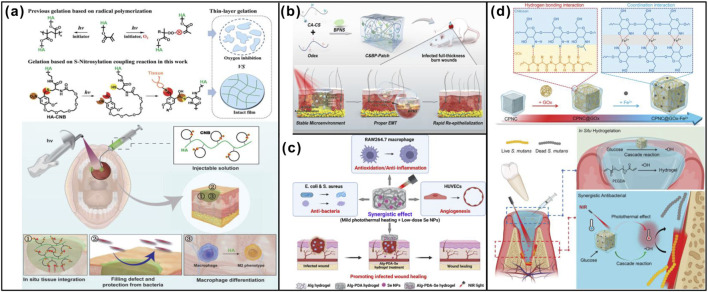
Light-responsive strategies synthetic polymer materials promote wound healing. **(a)** HA-CNB gels for promoting oral mucosa wound healing. Adapted from Ref. (Zhang et al., 2021), Copyright 2021, Wiley. **(b)** GF-free NIR-responsive adhesive C&BP-Patch intrinsically inducing proper EMT (partial EMT) to enable rapid re-epithelialization for full-thickness infectious burn wound healing. Adapted from Ref. (Wang X. et al., 2022), Copyright 2022, The Royal Society of Chemistry. **(c)** New kind of mild heat-assisted PDA/alginate hydrogels containing low-dose nanoselenium enhancing the healing rate of infected wounds. Adapted from Ref. (Xu et al., 2023), Copyright 2023, American Chemical Society. **(d)** CPNC@GOx-Fe^2+^ promoting bacteria-induced tooth-extraction wound healing. Adapted from Ref. (Chen et al., 2023), Copyright 2023, Wiley.

PDT involves the use of a photosensitizer, light of an appropriate wavelength, and oxygen. Upon light activation, the photosensitizer generates ROS that cause irreversible oxidative damage to microorganisms, achieving effective antibacterial treatment (Carrera et al., 2016). Wenjie Zhang et al. developed a light-responsive hydrogel named HA-CNB, based on cyclic o-nitrobenzyl-modified HA. This hydrogel undergoes an S-nitrosylation coupling reaction within seconds upon light exposure, leading to rapid gelation and strong adhesion to host tissues. It helps maintain a favorable local microenvironment by recruiting more MPO^+^ cells and M1 macrophages into the wound site, while also inducing M2 macrophage polarization, thereby significantly promoting wound healing (Figure 3a) (Zhang et al., 2021). Uislen B. Cadore et al. demonstrated that repeated PDT treatments can reduce bacterial load, suppress the expression of pro-inflammatory cytokines in immune cells, and attenuate local inflammatory responses, all contributing to improved wound healing outcomes (Cadore et al., 2019).

##### 4.1.1.2 PTT

PTT functions through the ability of photothermal materials to absorb energy from incident light (e.g., laser irradiation) and convert it into thermal energy. The localized release of heat enables the eradication of bacterial biofilms by disrupting the biofilm matrix and inducing thermal ablation of pathogens. Concurrently, the mild hyperthermia generated can stimulate angiogenesis and enhance blood vessel regeneration, thereby contributing to effective antibacterial treatment and accelerated wound healing (Jung et al., 2018).

Axel Meisgenier et al. applied a 445-nm blue laser for wound ablation and observed significantly smaller residual wound areas, reduced pain, and enhanced healing compared to conventional cold blade treatment. The shorter wavelength allows higher energy absorption by the target tissue, resulting in lower penetration depth, reduced backscattering, and minimized thermal side effects. The underlying mechanism may involve modulation of ROS production and influence on signaling pathways related to inflammation and regeneration, including interleukin-6 (IL-6), TGF-β, and fibroblast growth factor-2 (FGF-2) (Meisgei et al., 2023). Alper Sindel et al. experimentally confirmed that low-level laser therapy accelerates oral mucosal wound healing by expediting the inflammatory process, enhancing collagen synthesis, improving granulation tissue formation, stimulating fibroblast proliferation and growth factor secretion, increasing tissue perfusion, and inducing neovascularization (Özalp et al., 2022). Xiaomeng Wang et al. developed an NIR-responsive wound dressing (C&BP-Patch) using catechol-modified chitosan (CA-CS), oxidized dextran (Odex), and black phosphorus nanosheets (BPNSs). The CA-CS provided strong wet adhesion and absorbed exudates, rapidly forming an *in situ* hydrogel with Odex. Under 808 nm NIR irradiation, the patch generated localized heat for sterilization via photothermal effects, reduced inflammation, and promoted epithelial-mesenchymal transition (EMT), facilitating rapid epithelial cell migration and re-epithelialization (Figure 3b) (Wang X. et al., 2022). Xu Qing et al. designed a multifunctional composite hydrogel composed of polydopamine (PDA) and copper-doped calcium silicate ceramic (Cu-CS). The photothermal effect of the hydrogel enhanced the “thermo-ion effect” of copper ions, while PDA complexation with copper improved photothermal performance and cellular bioactivity. This system accelerated wound healing through antibacterial action, enhanced collagen deposition, and angiogenesis (Xu et al., 2020). Yingnan Liu et al. constructed a multilayer nanofiber patch with electrospun fiber layers sandwiching a phase-change material loaded with the photosensitizer indocyanine green (ICG) and low-molecular-weight fucoidan (LMWF). The top layer incorporated acellular dermal matrix. Under photothermal triggering, the patch released LMWF specifically targeting cancer cells without affecting oral mucosal keratinocytes, thereby promoting epithelial proliferation (Liu et al., 2022c). Jinfeng Xu et al. developed a photosensitive hydrogel (Alg-PDA-Se) based on polydopamine/alginate/nano-selenium, which offered controllable NIR photothermal properties. It released selenium nanoparticles to exert anti-inflammatory, pro-proliferative, pro-migratory, pro-angiogenic, and synergistic antibacterial effects, collectively enhancing wound healing (Figure 3C) (Xu et al., 2023). Yan-Yan Xie et al. constructed a novel hybrid hydrogel via self-assembly of N-(9-fluorenylmethoxycarbonyl)-L-phenylalanine (Fmoc-F) and berberine chloride (BBR), which exhibited aggregation-induced emission (AIE) behavior. Under daylight exposure, the hydrogel generated singlet oxygen and exerted photothermal effects, penetrating and disrupting biofilms while killing bacteria (Xie et al., 2021).

In summary, researchers have made significant strides in optimizing photothermal antibacterial therapy, yielding increasingly stable and favorable outcomes. However, challenges remain, including limited tissue penetration depth, potential thermal damage, and risks of inducing inflammatory responses. Further improvements are essential to advance clinical translation and therapeutic efficacy.

##### 4.1.1.3 Combined photodynamic and photothermal antibacterial therapy

The integration of photodynamic and photothermal therapies offers a promising synergistic strategy, leading to improved wound healing outcomes. This combined modality represents a compelling direction for further research and clinical development. Lei Chen et al. constructed a supramolecular photothermal cascade nanoreactor by employing a supramolecular strategy to modify palladium nanocubes (PdNCs) with chitosan, which exhibits strong near-infrared (NIR) absorption and high photothermal conversion efficiency. Through hydrogen bonding and coordination interactions between chitosan, glucose oxidase (GOx), and ferrous ions (Fe^2+^), GOx and Fe^2+^ were immobilized on the chitosan shell. This design significantly enhanced the thermal stability of GOx and its resistance to hydrolytic enzyme degradation. The nanoreactor demonstrated excellent photothermal performance, integrating PTT and chemodynamic therapy (CDT) into a supramolecular photothermal cascade system. This system drives the cascade reaction to generate hydroxyl radicals (·OH) by catalyzing glucose oxidation. The resulting ·OH and localized hyperthermia synergistically disrupt bacterial cell membrane integrity and function, increasing membrane fluidity and heterogeneity, thereby achieving potent antibacterial effects. Furthermore, the system facilitated *in situ* hydrogel formation via free radical polymerization and exerted selective antibacterial activity against oral microbial communities, enabling synergistic antimicrobial action and wound protection. This combined approach significantly accelerated wound healing (Figure 3d) (Chen et al., 2023).

#### 4.1.2 Temperature-responsive strategies

**FIGURE 4:**
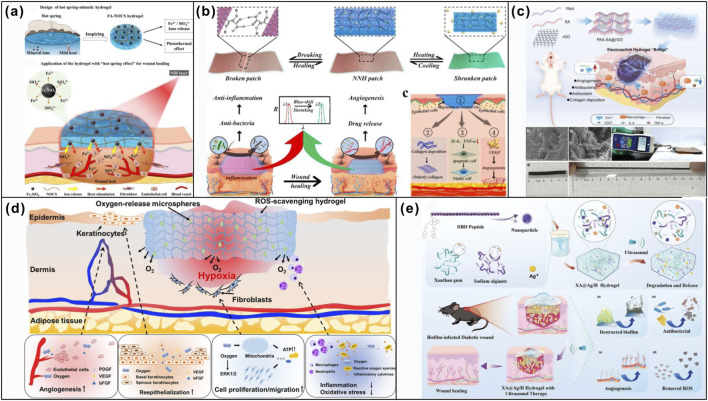
Externally triggered response strategies synthetic polymer materials promote wound healing. **(a)** The hot spring mimetic FA-NOCS hydrogel for wound healing application. Adapted from Ref. (Sheng et al., 2021), Copyright 2021, Elsevier. **(b)** The self-healing and temperature-responsive performances of NNH patches and their application to diabetic wound. Adapted from Ref. (Chen et al., 2022), Copyright 2022, Elsevier. **(c)** The PAA-SA@rGO hydrogel bridge creates a bioelectric “completed circuit” therapy accelerates the healing of oral mucosal wounds. Adapted from Ref. (Zhou et al., 2024), Copyright 2024, Wiley. **(d)** Mechanisms of accelerated wound healing by ORMs encapsulated in ROS-scavenging hydrogel. Adapted from Ref. (Guan et al., 2021), Copyright 2021, American Association for the Advancement of Science. **(e)** Ultrasound-responsive HBD peptide hydrogel for fast diabetic wound healing. Adapted from Ref. (Zong et al., 2024), Copyright 2024, Wiley.

The intraoral environment maintains a relatively high and stable temperature, which can be leveraged to achieve stimuli-responsive drug release using temperature-sensitive materials. Zahra Aliakbar Ahovan et al. developed a thermosensitive chitosan (CTS) hydrogel using chitosan and glycerol phosphate (β-GP). Through animal experiments, they demonstrated that this hydrogel combats extensively drug-resistant (XDR) bacteria caused by drug abuse and promotes complete healing of wounds infected with multidrug-resistant pathogens (Aliakbar Ahovan et al., 2020). Lili Sheng et al. fabricated a bioactive photothermal hydrogel based on iron zeolite and N,O-carboxymethyl chitosan. This system exhibits a “hot spring effect,” releasing bioactive ions under thermal stimulation while generating heat. The resulting thermo-ionic environment activates various angiogenic factors and signaling pathways, ultimately promoting angiogenesis and wound healing (Figure 4a) (Sheng et al., 2021). Andrew Padalhin et al. designed a temperature-responsive N-acetylcysteine hydrogel that enhances fibroblast activity and reduces intracellular ROS levels, thereby accelerating wound repair (Padalhin et al., 2024). Canwen Chen et al. constructed a temperature-sensitive poly (N-isopropylacrylamide) (PNIPAM) hydrogel with an inverse opal scaffold composed of N-acryloyl glycinamide (NAGA) and 1-vinyl-1,2,4-triazole (VTZ), loaded with vascular endothelial growth factor (VEGF). Under thermal stimulation, the hydrogel releases active substances, downregulates the expression of inflammatory factors, and promotes collagen deposition and angiogenesis, thus facilitating wound healing (Figure 4b) (Chen et al., 2022). Utilizing the relatively stable and elevated temperature of the oral cavity for responsive drug release represents a highly targeted and effective strategy for promoting wound healing, warranting further in-depth research.

#### 4.1.3 Magnetic field-responsive strategies

Magnetic fields offer significant advantages, including deep tissue penetration, minimal side effects, and high controllability (Zhang K. et al., 2022). Applied external magnetic fields can directly induce biological effects that promote wound healing (Nunes et al., 2021; Wang P. et al., 2025). For instance, electromagnetic fields enhance the expression of FGF-2, promoting endothelial cell proliferation and tubule formation, thereby inducing angiogenesis and accelerating wound healing (Khan and Arany, 2015). Devendra K. Agrawal et al. systematically discussed the impact of electromagnetic fields on the proliferation and migration of keratinocytes and fibroblasts, concluding that they improve wound healing by facilitating the migration and proliferation of fibroblasts, keratinocytes, and endothelial cells (Stojanovic et al., 2024). Furthermore, Léa Bedja‐Iacona MSc et al. utilized a Magnomega® device to deliver extremely low-frequency pulsed electromagnetic fields to human dermal fibroblasts. Their experiments confirmed that electromagnetic fields promote cell proliferation and fibroblast maturation, observations attributed to the activation of transmembrane Ca^2+^ ion fluxes involved in fibroblast migration and maturation (Bedja-Iacona et al., 2024). Peng Wang et al. developed a magnetically responsive nanocomposite hydrogel by bridging citric acid-coated ferrite nanoparticles (CFO NPs) with a polyvinyl alcohol (PVA) matrix using tannic acid (TA). This hydrogel exhibits enhanced mechanical properties and, under a static magnetic field (SMF), induces angiogenesis through mechanical stimulation, accelerating wound healing (Wang P. et al., 2022). While magnetic field-responsive strategies are undoubtedly effective, the necessity for an external magnetic field complicates their application, presenting significant challenges for clinical adoption.

#### 4.1.4 Electrical stimulation-responsive strategies

Exogenous electrical stimulation positively modulates the inflammatory, proliferative, and remodeling phases of wound healing by altering the electrical properties of the wound (Luo et al., 2021; Luo et al., 2023). By applying external electrical stimuli, microcurrent energy can reverse cell capacitance, facilitate the restoration of transepithelial potential (TEP), and promote wound healing (Khan and Arany, 2015). Camila Lopes Ferreira et al. demonstrated through animal studies that electrical stimulation enhances early palatal wound healing in mice by promoting the proliferation of epithelial and connective tissues and modulating the inflammatory response (Ferreira et al., 2021). Ruizeng Luo et al. proposed a novel approach using electrical stimulation as an adjunct therapy. They developed an electroactive dressing (EGD) that generates a safe, unidirectional pulsed electric field during negative pressure wound therapy (NPWT). This dressing alters the electric field within the wound, inducing a strong electrotactic response in epithelial cells and accelerating healing. Additionally, EGD promotes macrophage M2 polarization, further reducing healing time (Luo et al., 2023). Qiangqiang Zhou et al. developed a conductive polyacrylamide/sodium alginate crosslinked hydrogel composite incorporating reduced graphene oxide. This material acts as a bridge to regulate the “short-circuit” condition at the wound site and restore the endogenous electric field, thereby stimulating fibroblasts to produce growth factors and enhancing healing. Moreover, the composite upregulates genes associated with the ROS antioxidant signaling pathway, reducing oxidative damage. The incorporation of graphene provides excellent free radical scavenging and antioxidant properties, while graphene and its derivatives also exhibit anti-inflammatory effects (Figure 4c) (Zhou et al., 2024). Although electrical stimulation strategies have proven effective for wound healing, the requirement for an external power source places an additional burden on users, making ease of use a critical consideration for clinical translation.

#### 4.1.5 Oxygen stimulation-responsive strategies

Oxygen plays a critical role in wound healing, influencing processes such as cell proliferation, angiogenesis, immune response, and ECM formation. In the oral cavity, a dynamic environment with varying oxygen tensions, strategies that modulate oxygen delivery or respond to oxygen gradients offer promising therapeutic avenues for enhancing tissue repair. Ya Guan et al. developed a hydrogel containing oxygen-releasing microspheres that continuously release oxygen at the wound site. This hydrogel promotes epithelial regeneration, scavenges ROS, upregulates the expression of angiogenic growth factors, and downregulates pro-inflammatory cytokines, ultimately facilitating wound healing (Figure 4d) (Guan et al., 2021). Lin Jacob Varghese et al. evaluated the efficacy of topical ozone gel application on wound healing following mandibular third molar extraction. They found that ozone gel was superior to conventional treatment, demonstrating no toxicity, faster lesion resolution, symptom improvement, reduced postoperative pain and edema, enhanced wound healing, and lower dependence on opioid analgesics (Varghese et al., 2023). Mahshid Shabani et al. demonstrated through experiments that ozone accelerates the EMT of fibroblasts at the wound site, promoting healing (Shabani et al., 2024). Olga Di Fede et al. conducted a randomized clinical trial showing that ozone plays a significant role in both the inflammatory and proliferative phases of post-extraction wound healing. Ozone reduces inflammation and pain, stimulates healing through the synthesis of interleukins, leukotrienes, and prostaglandins, and overall enhances wound recovery (Di Fede et al., 2024).

While oxygen-releasing microspheres represent a relatively effective and convenient method, related research remains limited, and their efficacy requires further validation. Ozone therapy, administered via external application, cannot be sustained for prolonged periods, necessitating further development of controlled-release technologies.

#### 4.1.6 Ultrasound-responsive strategies

Both high-frequency and low-frequency ultrasound have been demonstrated to promote wound healing. Techniques such as saline aerosolization and non-contact low-frequency mist therapy can stimulate cells in the wound bed to secrete more growth factors, increase collagen synthesis, reduce inflammation, and induce vasodilation, thereby enhancing the healing of oral soft tissues. Low-intensity pulsed ultrasound has been shown to promote fracture healing through the release of bFGF and transforming growth factor, and may also facilitate soft tissue repair (Toma et al., 2021). The team led by Lanlan Zong developed an ultrasound-responsive hydrogel incorporated with heparin-binding domain peptide nanoparticles. The N-terminus of the HBD peptide was further modified with hydrophobic Nap-FF(K), resulting in the sequence Nap-FF(K)YIGLKDRKRPSELRRIASQVKYA, enabling self-assembly into nanoparticles. Xanthan gum and sodium alginate (SA) were pre-mixed with silver ions and HBD peptide nanoparticles to facilitate cross-linking. Subsequent addition of Ca^2+^ ions triggered cross-linking, forming a robust 3D dual-network scaffold (XA@Ag/H hydrogel). Under ultrasound stimulation, the hydrogel released xanthan gum, which actively neutralized ROS, alleviating oxidative stress in the diabetic wound environment. Additionally, ultrasound exposure fragmented the hydrogel, enabling deep penetration and delivery of nanoparticles into the wound tissue, significantly promoting healing (Figure 4e) (Zong et al., 2024). While promising, this therapeutic approach requires further investigation before clinical application.

#### 4.1.7 Pressure stimulation-responsive strategies

The accumulation of interstitial fluid in wounds can significantly impede the healing process. Applying targeted pressure can enhance venous reabsorption of excess fluid and improve the wound microenvironment. Research has demonstrated that NPWT accelerates healing by promoting the migration of leukocytes and fibroblasts into the wound area, which in turn secrete growth factors that improve the healing environment (Khan and Arany, 2015).

### 4.2 Internally triggered response strategies

Wound formation induces changes in the physical and/or biochemical characteristics of the local microenvironment. Synthetic polymer materials can be engineered to respond to these internal triggers, exerting corresponding biological effects on cells or tissues to promote wound healing.

#### 4.2.1 Enzyme-responsive strategies

**FIGURE 5:**
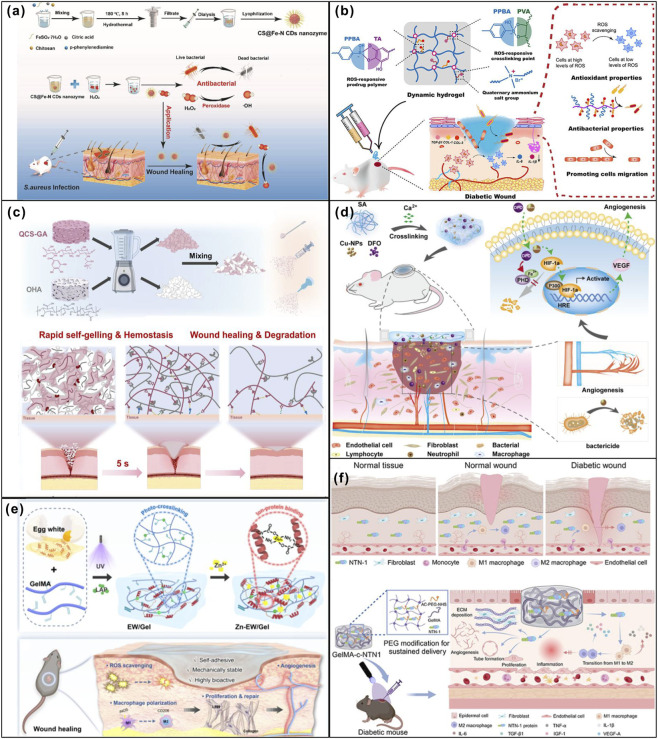
Internally triggered response strategies synthetic polymer materials promote wound healing. **(a)** The antibacterial ability of CS@Fe-N CDs nanozyme and its wound healing properties. Adapted from Ref. (Li L. et al., 2024), Copyright 2024, Elsevier. **(b)** Multistage ROS-Responsive and Natural Polyphenol-Driven Prodrug Hydrogels for Diabetic Wound Healing. Adapted from Ref. (Ni et al., 2022), Copyright 2022, American Chemical Society. **(c)** QCS-GA + OHA self-gelling hemostatic microsheets for emergency hemostasis and wound repair. Adapted from Ref. (Shan et al., 2025), Copyright 2025, Elsevier. **(d)** The treatment strategy for diabetic wound healing using the bioactive hydrogel composite containing DFO and Cu-NPs. Adapted from Ref. (Li et al., 2022), Copyright 2022, Elsevier. **(e)** The Zn-EW/Gel functional hydrogel dressing accelerated wound healing. Adapted from Ref. (Li et al., 2025), Copyright 2025, Elsevier. **(f)** Netrin-1 co-crosslinked hydrogel accelerated diabetic wound healing *in situ* by modulating macrophage heterogeneity and promoting angiogenesis. Adapted from Ref. (Shu et al., 2024), Copyright 2024, Elsevier.

Enzymes are essential natural macromolecules that catalyze biochemical reactions in living organisms and play a crucial role in wound healing. For example, MMPs can degrade ECM growth factors; abnormal MMP levels can impede the healing process. Some nanozymes with peroxidase-like activity can accumulate excessive ROS, disrupting bacterial cell structures and exerting antibacterial effects. Lanling Li developed a novel Fe/N-doped chitosan-chelated carbon dot-based nanozyme (CS@Fe-N CDs) that converts hydrogen peroxide (H_2_O_2_) into ·OH, disrupting ROS balance and bacterial integrity, ultimately achieving bacteriostatic or bactericidal effects (Figure 5a) (Li L. et al., 2024). Ferreira CC et al. designed a semi-interpenetrating network hydrogel composed of poly (Ferreira et al., 2025) and chitosan, which served as matrices for controlled release of papain, a proteolytic enzyme with healing and debridement properties, combining antibacterial and pro-regenerative mechanisms to accelerate wound healing (Ferreira et al., 2025).

#### 4.2.2 ROS-responsive strategies

ROS, including H_2_O_2_, superoxide, ·OH, and other oxygen metabolites, accumulate at wound sites. Excessive ROS enhances oxidative stress and delays healing. Therefore, materials designed to scavenge ROS can facilitate wound recovery. Chenxi Tu et al. designed a multifunctional hydrogel using super-distributed L-lysine-modified manganese dioxide nanozymes as cross-linkers, further loaded with pravastatin sodium to participate in nitric oxide (NO) synthesis. This hydrogel cleared various types of ROS, generated O_2_, killed broad-spectrum bacteria, and protected cells from oxidative stress. It also reduced neutrophil infiltration, enhanced M2 macrophage polarization, lowered pro-inflammatory cytokine levels, and elevated anti-inflammatory cytokine levels, collectively promoting wound healing (Tu et al., 2022). Jiwei Sun et al. developed a Fe^3+^-tannic acid (Fe-TA) complex-modified P (AM-AA) hydrogel (Fe-TA@P (AM-AA)) that stably adhered to and protected oral wounds. By releasing tannins through reaction with environmental ROS via polyphenol groups, it induced M2 macrophage polarization and reduced neutrophil infiltration, significantly rebalancing neutrophils and M1/M2 macrophages to remodel the microenvironment and promote healing (Sun et al., 2023). Zhipeng Ni et al. prepared a nanoparticle hydrogel by mixing TA, phenylboronic acid-modified polyphosphazene, and PVA. It effectively scavenged free radicals *in vitro*, promoted cell migration, downregulated pro-inflammatory cytokines, and upregulated the gene expression of transforming growth factor, thereby facilitating re-epithelialization, reducing ROS, alleviating inflammation, and accelerating wound healing (Figure 5b) (Ni et al., 2022). Wen Fang et al. fabricated an injectable hydrogel using CQCS and DB-PEG2000. Its strong adhesion and physical sealing promoted hemostasis, while enhancing collagen deposition, angiogenesis, and ROS clearance to shorten the inflammatory phase and accelerate healing (Fang et al., 2023). Yu Miao et al. synthesized a boronic acid molecular cross-linker (SPBA) from succinic acid and 4-(bromomethyl)phenylboronic acid, which formed borate ester bonds with diols on natural polysaccharide SA to prepare a pH/ROS dual-responsive hydrogel (SA-SPBA) loaded with *Lactobacillus rhamnosus* (L.rha). The borate ester bonds endowed the hydrogel with ROS-scavenging ability, reducing excessive ROS and inflammation. L.rha exerted antibacterial effects by producing organic acids, while the released succinic and boronic acids also contributed to antimicrobial activity (Miao et al., 2024).

#### 4.2.3 pH-responsive strategies

The normal human microenvironment is weakly alkaline (pH ≈ 7.4), while wound sites exhibit a slightly acidic pH, typically 0.5–1 unit lower than that of surrounding healthy tissue (Karimi et al., 2016). This acidic microenvironment can be leveraged to design pH-sensitive materials for responsive wound therapy. Tao Lin et al. developed an intelligent composite hydrogel using SA, carboxymethyl chitosan, and quaternized chitosan as the backbone, loaded with a triple-drug combination (gemcitabine, a GABAB receptor agonist, and a JAK3 inhibitor). This hydrogel featured a dense honeycomb structure conducive to drug loading and wound ventilation. It demonstrated antibacterial and anti-inflammatory effects by reducing TNF-α and IL-6 levels in inflammatory cells, significantly promoting healing in a rat oral ulcer model. Moreover, the hydrogel exhibited dual pH sensitivity with varying swelling behaviors under different pH conditions, making it adaptable to dynamic oral environments (Lin et al., 2023). Meng Wang et al. synthesized honokiol-grafted chitosan hydrochloride and crosslinked it with genipin to form a pH-responsive hydrogel. This system synergistically inhibited bacteria and scavenged ROS, modulating inflammation and infection to accelerate wound healing (Wang M. et al., 2022). Chenggui Wang et al. prepared a hydrogel loaded with exosomes derived from adipose mesenchymal stem cells. In the acidic wound environment, the hydrogel released exosomes that promoted proliferation, migration, and tube formation of human umbilical vein endothelial cells (HUVECs), enhancing angiogenesis and re-epithelialization (Wang et al., 2019).

#### 4.2.4 Fluid-responsive strategies

Residual blood at wound sites can hinder dressing adhesion and delay healing. Therefore, dressings that directly absorb blood and promote hemostasis are highly desirable. Mai El Halawany developed a tranexamic acid (TXA)-loaded sodium alginate/nano-hydroxyapatite (SA/Nano-HA) composite aerogel. This material significantly reduced clotting and recalcification times, showing promise as a post-extraction hemostatic dressing. Alginate contributed to hemostasis through high absorbency and hydrophilicity, TXA inhibited fibrinolysis by preventing plasminogen conversion to plasmin and promoted platelet aggregation; alginate also formed a rigid gel upon crosslinking with calcium ions, activating platelets and clotting factors. Nano-HA facilitated cell adhesion and proliferation and contributed to hemostasis via calcium release. However, despite excellent hemostatic performance, the aerogel led to slower wound healing, possibly due to TXA-induced cell detachment delaying cell migration (El Halawany et al., 2022). Yingli Shan et al. designed a hydrogel composed of quaternized chitosan-grafted-gallic acid (QCS-GA) and oxidized HA. QCS-GA provided antibacterial, pro-coagulant, and tissue-adhesive properties, while OHA formed chemical crosslinks with tissues via its aldehyde groups. The hydrogel rapidly self-gelled into a dense porous structure, effectively absorbing blood and providing mechanical support to accelerate wound healing (Figure 5c) (Shan et al., 2025).

#### 4.2.5 Cytokine response strategy

Numerous cytokines play crucial roles in cell proliferation, migration, and angiogenesis. In response to this phenomenon, promoting vascular regeneration and re-epithelialization and ultimately wound healing can be achieved by delivering cytokines or directly stimulating their production.

Shengbo Li et al. developed a SA hydrogel cross-linked with calcium ions, incorporating deferoxamine (DFO) and copper nanoions (SA-DFO/Cu). This hydrogel promotes vascular regeneration by stimulating the expression of hypoxia-inducible factor 1α (HIF1α) and VEGF. It also exhibits antibacterial effects against *E. coli* and *Staphylococcus aureus*, thereby enhancing diabetic wound healing (Figure 5d) (Li et al., 2022). Yannan Li et al. fabricated a nanocomposite hydrogel scaffold using poly (ethylene glycol) diacrylate (PEGDA), copper-containing bioactive glass nanoparticles and sodium alginate. This scaffold facilitates the repair of vascular networks and promotes wound healing by upregulating HIF-1α and VEGF expression and enhancing collagen matrix deposition (Li et al., 2020). Nan Zhao et al. created an aptamer-fibrinogen hydrogel that promotes angiogenesis and wound healing by releasing and stimulating VEGF expression (Zha et al., 2019). Nasrul Wathoni et al. prepared an SAC (a natural macromolecular polysaccharide) hydrogel film containing keratinocyte growth factor (KGF) (SAC/KGF-HF), which accelerates wound re-epithelialization by promoting fibroblast migration and stimulating fibroblasts and epithelial cells, thus improving wound healing (Wathoni et al., 2019). Qiangqiang Zhou et al. designed a nanocarrier system composed of tetrahedral DNA nanostructures (TDN) and miR-132. TDN exhibits excellent cellular uptake and drug delivery capabilities, efficiently delivering miR-132 into cells to regulate the proliferation and migration of human oral mucosal fibroblasts and HUVECs, while modulating inflammatory and antioxidant processes. The miR@TDN system effectively and stably delivers miR-132 to damaged mucosa, significantly enhancing cellular repair functions and accelerating wound healing by regulating inflammatory responses, promoting vascular regeneration, and strengthening antioxidant defense mechanisms (Zhou et al., 2025). López-Domínguez S et al. developed a hydrogel derived from cellulose extracted from agave bagasse. The cellulose provides a biocompatible scaffold that supports cell proliferation and tissue regeneration, promotes angiogenesis and fibrogenesis, and ultimately facilitates the repair and regeneration of gingival connective tissue and wound healing (López-Domínguez et al., 2025). Patole V et al. designed a polyelectrolyte complex film composed of gallic acid-modified guar gum (GA-GG) and chitosan (CS). GA-GG provides antioxidant and antibacterial properties, while CS promotes cell proliferation and tissue regeneration. This composite film accelerates wound healing by offering an antibacterial, antioxidant, and cell-proliferation-supportive microenvironment (Patole et al., 2025). Jie Zhao et al. synthesized 13 Trolox amides and 7 Trolox esters to identify novel antioxidants. Among them, cyclobutyl 6-hydroxy-2,5,7,8-tetramethylchroman-2-carboxylate was found to protect HaCaT cells from oxidative stress, inflammation, and cellular damage by activating the Nrf2/HO-1 signaling pathway and inhibiting the NF-κB pathway, thereby promoting wound healing (Zhao et al., 2025).

#### 4.2.6 Ion response strategy

Eduard Ferrés-Amat et al. conducted a randomized double-blind study involving 81 patients and evaluated a novel bone bioactive liquid (BBL). This solution contains calcium chloride (CaCl_2_) and magnesium chloride hexahydrate (MgCl_2_·6H_2_O), which carry a net negative charge, creating an environment that allows active ions to interact with plasma, progenitor endothelial cells, and epithelial cells, effectively promoting soft tissue regeneration (Ferrés-Amat et al., 2022). Jingfeng Yuan et al. synthesized an antibacterial hydrogel using PEG diglycidyl ether and ε-poly-L-lysine, wherein the cationic peptide ε-poly-L-lysine exerts bactericidal effects (Yuan et al., 2021). Li Q et al. designed a natural protein-based multifunctional hydrogel dressing composed of egg white (EW) and zinc ions (Zn^2+^). The EW component rapidly photopolymerizes to form a 3D network structure, providing excellent biocompatibility and mechanical properties. Zn^2+^ enhances the hydrogel’s adhesion and antibacterial performance through coordination with proteins. The combined action of functional proteins and active ions promotes fibroblast proliferation, type I collagen expression, and angiogenesis, ultimately accelerating wound healing (Figure 5e) (Li et al., 2025).

#### 4.2.7 Gene target response strategy

Yuqi He et al. investigated the use of novel nanodrug carriers for treating recurrent oral ulcers, demonstrating significant efficacy and low recurrence rates. For instance, gene-specific nanodrug carriers utilize targeted delivery systems to transport therapeutic genes into relevant tissues, enabling the expression of large quantities of proteins beneficial for treatment (He et al., 2021). However, gene-targeted therapies still face challenges related to safety and stability, including off-target effects, drug resistance, high development costs, and limited applicability across populations. Further research is needed to address these issues and refine the approach. Future directions may include multi-target combination therapies and personalized precision medicine.

#### 4.2.8 Immune response strategy

Fu Tingshu et al. encapsulated recombinant netrin-1 protein (a diffusible laminin-like secreted protein) into GelMA hydrogel for sustained release. Experiments showed that netrin-1 regulates macrophage polarization via the A2bR/STAT/PPARγ signaling pathway and mildly promotes endothelial cell proliferation, thereby enhancing wound healing (Figure 5f) (Shu et al., 2024). Andrew M. Overmiller et al. found that in diabetic mouse models, the transcription factor FOXM1 is pharmacologically inhibited, and its target genes are dysregulated in chronic wounds, leading to suppressed activation, recruitment, and survival of immune cells. Thus, targeting the FOXM1 pathway and its downstream genes may offer novel therapeutic strategies to reprogram chronic non-healing wounds into acute healing wounds (Overmiller et al., 2022).

### 4.3 Multi-stimuli responsive strategies

For complex wound conditions, combination therapies, the integrated application of multiple therapeutic approaches, are increasingly employed. Numerous studies have reported multi-stimuli responsive strategies that can simultaneously respond to various external or internal triggers, achieving enhanced pro-healing therapeutic effects.

#### 4.3.1 Dual- and multi-stimuli responsive strategies from the internal microenvironment

##### 4.3.1.1 Dual internal stimuli responsive combination therapies

**FIGURE 6:**
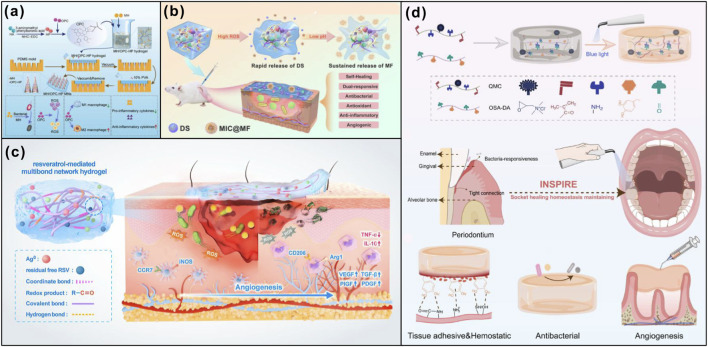
Multiple internal stimuli responsive strategies synthetic polymer materials promote wound healing. **(a)** ROS-responsive gel-based microneedle patches promote oral ulcer wounds healing. Adapted from Ref. (Zhang X. et al., 2025), copyright 2025, American Chemical Society. **(b)** A pH/ROS dual responsive injectable glycopeptide hydrogel accelerated wound repairing. Adapted from Ref. (Wu et al., 2022), copyright 2022, Elsevier. **(c)** Resveratrol-mediated multibond network hydrogel with efficient sustainable antibacterial, ROS scavenging, pro-angiogenesis, and immunomodulation activities for accelerating infected wound healing. Adapted from Ref. (Feng et al., 2025), copyright 2025, American Chemical Society. **(d)** QDL hydrogel oromotes full-course socket healing. Adapted from Ref. (Guo et al., 2024), copyright 2024, American Chemical Society.

Combining ROS and Immune Responses: By reducing excess ROS and oxidative stress while synergistically regulating immune cell functions, this approach efficiently clears pathogens and enhances the body’s innate defense capabilities. Siyuan He et al. developed oral ulcer tissue-adhesive nanoparticles via the esterification reaction of polyglutamic acid and tannic acid. TA, rich in phenolic hydroxyl groups that are easily oxidized by free radicals, scavenges excess ROS and reduces oxidative stress to promote wound healing. Doxycycline hydrochloride (DCH) was incorporated into the nanoparticles; it exerts antibacterial effects by altering bacterial membrane permeability and interfering with protein synthesis. Additionally, these nanoparticles induce cytokine production, influencing macrophage polarization toward the M2 phenotype for immunomodulation (He et al., 2023). Xuancheng Zhang et al. designed a polymer gel formed by dynamic covalent borate bonds between oligomeric proanthocyanidins and 3-(aminomethyl) phenylboronic acid-modified HA, loaded with minocycline hydrochloride to create MH/OPC-HP microneedle patches with ROS-responsive characteristics. The OPC component responds to ROS, enabling sustained release to scavenge excess ROS, regulate immune responses, promote M2 macrophage polarization, and suppress inflammatory cytokine expression, ultimately accelerating oral ulcer healing (Figure 6a) (Zhang X. et al., 2025).

Combining Immune and Cytokine Responses: This strategy intelligently modulates the balance between pro-inflammatory factors (e.g., low expression of IL-6, TNF-α) and regenerative factors (e.g., high expression of COL-Ⅰ, VEGF), synergistically reducing inflammatory infiltration, suppressing aberrant immune responses, and actively promoting collagen deposition and vascular regeneration to efficiently remodel the healing microenvironment and accelerate tissue repair and regeneration. Siyu Li et al. designed a reversibly adhesive hydrogel and demonstrated in animal experiments that it effectively reduces inflammatory infiltration, promotes collagen deposition, and enhances vascular regeneration. The hydrogel’s adhesion is strengthened through radical copolymerization of cationic monomer trimethylammonium chloride (ATAC), hydrophobic monomer ethylene glycol phenyl ether acrylate (PEA), and N-isopropylacrylamide (NIPAAm), along with electrostatic interactions and hydrogen bonding. It exhibits excellent anti-swelling properties and antibacterial efficiency. By downregulating IL-6 and TNF-α and upregulating COL-Ⅰ and VEGF, it exerts anti-inflammatory effects, protects thrombi, promotes collagen deposition, and facilitates vascular and bone regeneration, thereby accelerating the healing of tooth extraction wounds (Li S. et al., 2024).

Combining ROS and Cytokine Responses: This approach directly scavenges ROS and inhibits its generation to alleviate oxidative stress, while activating pro-angiogenic signaling pathways to synergistically optimize the repair microenvironment, thereby efficiently reducing inflammation and accelerating tissue regeneration and wound healing. Huan Lei et al. utilized paramylon secreted by *Euglena gracilis* to prepare a hydrogel that suppresses ROS generation by directly neutralizing ROS and chelating metal ions required for ROS formation. Simultaneously, it promotes vascularization via the HIF-1α-VEGF pathway, effectively reducing wound inflammation and facilitating wound repair (Lei et al., 2022).

Combining Ion and Cytokine Responses: Leveraging ion effects, this strategy synergistically promotes VEGF secretion, enhances vascular regeneration, and reduces inflammation to optimize the healing microenvironment and accelerate tissue repair and wound closure. Hongjuan Weng et al. developed an N-succinyl chitosan oxidized HA (NSC-OHA) hydrogel incorporated with calcium ions (Ca^2+^) and/or tetra-armed amino-terminated polyethylene glycol (4-Arm-PEGNH_2_). Chitosan achieves hemostasis by absorbing negatively charged red blood cells and exhibits broad-spectrum bactericidal activity. HA absorbs blood and wound exudates while promoting cell proliferation, migration, and epidermal regeneration. Additionally, calcium ions, as coagulation factor IV, improve hemostasis, stimulate VEGF secretion, promote vascular regeneration, and reduce inflammation, ultimately facilitating wound healing (Weng et al., 2022).

Combining pH and ROS Responses: This strategy triggers drug release in acidic environments and scavenges ROS to mitigate oxidative damage, synergistically inhibiting inflammation, promoting collagen deposition, and enhancing angiogenesis to effectively optimize the microenvironment of refractory wounds and accelerate tissue regeneration and healing. Ye Wu et al. constructed a pH/ROS dual-responsive hydrogel based on phenylboronic acid-grafted oxidized dextran and caffeic acid-grafted ε-polylysine, embedded with mangiferin (MF) and diclofenac sodium (DS). This hydrogel promotes wound healing in acidic environments by suppressing inflammation, enhancing collagen deposition, and stimulating angiogenesis (Figure 6b) (Wu et al., 2022).

Combining pH and Enzyme Responses: Triggered by weakly alkaline and enzymatic environments, this approach enables intelligent drug release and material degradation, synergistically inhibiting bacterial growth, disrupting biofilm structures, and optimizing the wound microenvironment to effectively promote the healing of infected wounds. Sijie Zhou et al. prepared a HA-histamine (His)/Zr^4+^ metal ion hydrogel with pH and enzyme responsiveness. Under weakly alkaline conditions and hyaluronidase action, it promotes wound healing by inhibiting bacterial growth and biofilm formation (Zhou et al., 2023).

Combining ROS Response and Immunomodulation: This strategy efficiently clears excess ROS to alleviate oxidative stress while synergistically regulating immune cell functions and promoting angiogenesis, thereby clearing pathogens, optimizing the inflammatory microenvironment, and accelerating infected wound healing. Feng J et al. designed a multi-bond network hydrogel mediated by resveratrol (RSV), which provides antibacterial, ROS-scavenging, angiogenic, and immunomodulatory functions to ultimately accelerate the healing of infected wounds (Figure 6c) (Feng et al., 2025).

Combining Ion Response and Antibacterial Function: Through ion exchange, this approach precisely controls the release of silver ions for efficient and sustained bactericidal effects, effectively suppressing wound infection and promoting healing. Chan Wang et al. developed an AgNPs-Alg composite material where sodium ions facilitate the ion exchange of alginate to release silver ions from the complex for antibacterial action and wound healing promotion. However, further research is needed to optimize its performance *in vivo* (Wang et al., 2014).

##### 4.3.1.2 Triple internal stimuli responsive combination therapies

Combining Ion Response, Cytokine Response, and Gene Target Response: This strategy leverages ion-mediated rapid hemostasis and antibacterial effects, synergistically upregulating pro-healing genes and signaling pathways to promote angiogenesis and epithelial migration, thereby efficiently optimizing the wound microenvironment and accelerating tissue regeneration and wound healing. Yuxuan Guo et al. used a dual-crosslinking method to crosslink quaternized methacryloyl chitosan (QMC) with dopamine-grafted oxidized sodium alginate (OSA-DA), creating a QDL hydrogel as a clinical tooth extraction wound dressing. The hydrogel exhibits excellent mechanical strength, self-healing properties, and robust adhesion. Its inherent positive charge aggregates negatively charged red blood cells to promote clot formation and achieve rapid hemostasis. The functional groups trap and kill bacteria, effectively inhibiting the growth of *Staphylococcus aureus* and *E. coli*. By upregulating the expression of wound healing-related genes and signaling pathways, it promotes epithelial migration and neovascularization, accelerating wound healing (Figure 6D) (Guo et al., 2024). In summary, multiple internal stimuli responsive strategies and materials (e.g., nanoparticles, hydrogels) achieve precise regulation through mechanisms such as ROS scavenging, antibacterial action, immunomodulation, hemostasis, anti-inflammation, and pro-angiogenesis. By integrating responses to pH, ROS, enzymes, and other stimuli, these strategies synergistically address diverse wound problems, enhance healing outcomes, and provide efficient solutions for various types of trauma repair.

#### 4.3.2 Dual- and multi-stimuli responsive sstrategies involving external triggers

##### 4.3.2.1 Dual external stimuli responsive combination therapies

Combining Electrical and Magnetic Responses: This approach utilizes external electromagnetic field stimulation to activate fibroblast migration and regulate the secretion of key cytokines, synergistically optimizing cellular behavior and the microenvironment, thereby non-invasively promoting tissue regeneration and wound healing. Erica Costantini et al. demonstrated through experiments that pulsed radiofrequency electromagnetic fields promote wound healing by activating fibroblast migration and modulating cytokine production (Costantini et al., 2024).

**FIGURE 7:**
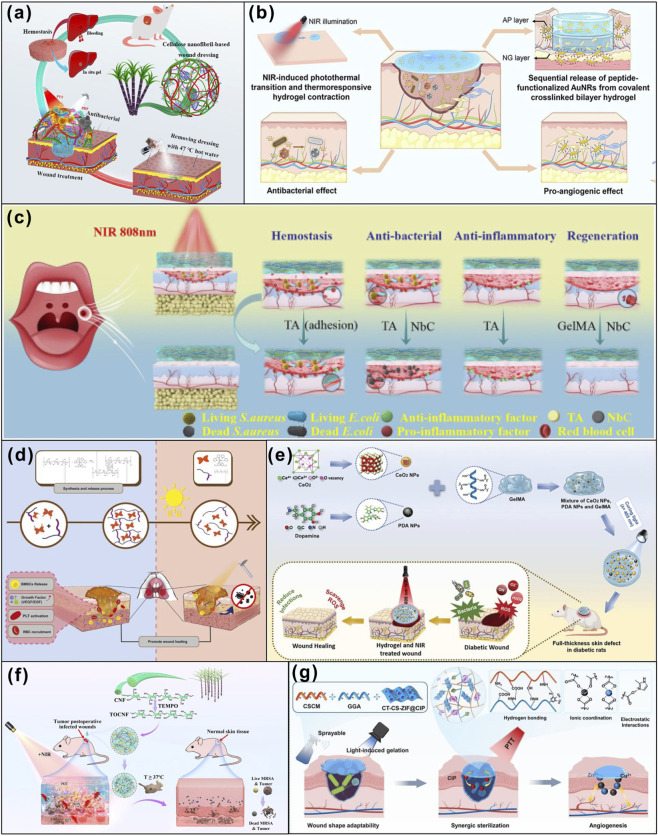
Multiple external stimuli responsive strategies synthetic polymer materials promote wound healing. **(a)** A white light and NIR dual light-responsive cellulose nanofibril (CNF)-based *in situ* hydrogel wound dressing promotes wound healing. Adapted from Ref. (Shi et al., 2022), copyright 2022, Elsevier. **(b)** AuP-AP/NG bilayer hydrogels promote diabetic wound healing. Adapted from Ref. (Huang et al., 2023), copyright 2023, Elsevier. **(c)** Illustration of the mechanism of NbC/TA–GelMA hydrogel with multifunctional properties for potential application in oral mucosal impairment. Adapted from Ref. (Chen et al., 2024), copyright 2024, Royal Society of Chemistry. **(d)** DCS-RuB2A2-BMSCs hydrogel Promote oral mucosal wound healing. Adapted from Ref. (Qi et al., 2023), copyright 2023, Elsevier. **(e)** NIR-responsive GelMA/CeO_2_/PDA hydrogelpromote diabetic wound healing. Adapted from Ref. (Xue et al., 2025), copyright 2025, Wiley. **(f)** The multifunctional pH, temperature, and NIR multiple responsive bioinspired CNF-based *in situ* liquid wound dressing can effectively heal wound. Adapted from Ref. (Zhang et al., 2020), copyright 2020, Ivyspring International Publisher. **(g)** Chitosan and gelatin based sprayable hydrogels for preventing wound secondary injury and infections effectively. Adapted from Ref. (Wang F. et al., 2025), copyright 2025, Elsevier.

Combining Light and Temperature Responses: This strategy leverages the photothermal effect to achieve precise temperature-controlled sterilization and drug release, while synergistically regulating the expression of thermosensitive genes and protein activity. This allows for intelligent modulation of the microenvironment, efficient pathogen clearance, and accelerated tissue repair. Xiaokun Shi et al. prepared a temperature-responsive *in situ* hydrogel based on dual white light and NIR light-sensitive fibers (CNF). This hydrogel uses white light nanofibers and endogenous antibacterial nanofibers as the scaffold, with Prussian blue nanoparticles, Pluronic® F127, and hydroxypropyl methylcellulose serving as the NIR sensitizer, thermosensitive switch, and adhesive, respectively. The dressing can rapidly absorb blood and form an *in situ* gel as a physical barrier, while also exhibiting antibacterial effects to promote wound hemostasis and healing. Furthermore, it enables drug release in response to temperature changes without causing secondary damage during dressing changes (Shi et al., 2022) (Figure 7a). Xiaoqiong Huang et al. developed a bilayer structured hydrogel using peptide-functionalized gold nanorods (AuNRs). It consists of a highly temperature-responsive poly (N-isopropylacrylamide)/gelatin methacryloyl (NG) thermosensitive layer and a highly stretchable alginate/polyacrylamide (AP) non-responsive layer. Under NIR irradiation, the hydrogel demonstrates enhanced bactericidal effects while also accelerating the proliferation, migration, and tube formation of fibroblasts and endothelial cells, promoting angiogenesis and collagen deposition, thereby facilitating wound healing (Huang et al., 2023) (Figure 7b). In summary, dual external stimuli responsive combination therapies involve the rational design of material structures and functions to integrate the regulatory mechanisms of different stimuli. This approach not only addresses multiple challenges in wound healing (such as hemostasis, infection, and slow tissue regeneration) in a targeted manner but also enhances the overall therapeutic efficacy through synergistic effects, offering more efficient strategies for the repair of complex wounds.

#### 4.3.3 Hybrid stimuli-responsive strategies: integrating internal and external cues

##### 4.3.3.1 Dual stimuli responsive combination therapies

Combining Magnetic and Cytokine Responses: This strategy utilizes magnetic fields to guide targeted cell colonization and control the release of regulatory factors, synergistically promoting the secretion of pro-angiogenic cytokines and the formation of vascular networks. This efficiently remodels the regenerative microenvironment, accelerating tissue repair and wound healing. Miyeon Noh et al. prepared an alginate/poly-L-ornithine/gelatin hydrogel sheet (MPS) with grooved patterns and embedded magnetic nanoparticles (MNPs). Endothelial progenitor cells were delivered onto the hydrogel sheet, promoting their colonization on the wound and secretion of pro-angiogenic cytokines to induce vascular regeneration, thereby facilitating wound healing (Noh et al., 2019). Radha Daya et al. developed a HA hydrogel containing curcumin-coated magnetic nanoparticles. It promotes angiogenesis by stimulating VEGF secretion, ultimately enhancing wound healing (Daya et al., 2022).

Combining Light and Cytokine Responses: This approach leverages the photothermal effect for precise sterilization and regulation of angiogenic factor expression, synergistically promoting anti-inflammatory effects, cell proliferation, and tissue regeneration. This efficiently clears pathogens and accelerates wound healing. Jiayuan Chen et al. designed a niobium carbide (NbC)/TA-GelMA hydrogel system. This hydrogel adheres well in the moist oral environment. NbC promotes wound healing through photothermal antibacterial effects and pro-angiogenesis, while TA controls bleeding effectively through anti-inflammatory and astringent effects, which contract tissues and reduce vascular permeability, reducing the damaged area to promote healing (Chen et al., 2024) (Figure 7c). Congyang Mao et al. fabricated a hybrid hydrogel based on electrostatic interactions between two-dimensional BPNSs and chitosan. Under visible light irradiation, BP generates singlet oxygen, damaging cell membranes, proteins, and DNA to exert bactericidal effects. Simultaneously, BP enhances fibrinogen formation, accelerates scabbing, triggers the PI3K/Akt and ERK1/2 signaling pathways, enhances cell proliferation and differentiation, and promotes bacteria-associated wound healing (Mao et al., 2018). Shun Yao et al. developed a novel microneedle (MN) patch combining a porous metal-organic framework (MOF) of NO-loaded copper benzene-1,3,5-tricarboxylate wrapped in graphene oxide (GO). The GO shell provides a NIR photothermal effect, enabling precise, deep release of NO into the wound under NIR irradiation, promoting tissue regeneration and collagen deposition, which benefits wound healing (Yao et al., 2022).

Combining Light and Immune Responses: This strategy utilizes light-controlled drug release and photothermal effects for precise sterilization, while synergistically regulating immune cell phenotype polarization and suppressing excessive inflammation. This efficiently clears pathogens, optimizes the regenerative microenvironment, and accelerates wound healing. Wenxin Qi et al. constructed a light-responsive antibacterial hydrogel containing sustained-release bone marrow mesenchymal stem cells (BMSCs) using bipyridyl Ru-DCs. Under light conditions, RuB2A photolysis triggers hydrogel detachment, resulting in hydrogel degradation alongside antibacterial action. The hydrogel, based on dodecyl chitosan, is loose and porous, provides excellent hemostasis and coagulation, and activates platelets to promote healing. Furthermore, BMSCs can migrate to the injury site *in vivo* and accelerate repair through immunomodulation and growth factor production, promoting neovascularization, re-epithelialization, and stimulating angiogenesis (Qi et al., 2023) (Figure 7d). Lin Jin et al. prepared a NIR-responsive bandage (MNFs@V–H@DA) composed of a core of MXene-loaded nanofibers (MNF) and a shell of dopamine-HA hydrogel, encapsulating growth factor (VEGF, abbreviated V) and an H_2_S donor (diallyl trisulfide, DATS, abbreviated DA). The abundant MXene nanosheets in the fiber skeleton the bandage excellent photothermal properties. PTT promotes wound healing and controls VEGF release to prevent excessive angiogenesis. Simultaneously, H_2_S release induces macrophage polarization towards an M2-like phenotype, regulating the immune microenvironment and suppressing excessive inflammation at the wound site, favoring healing (Jin et al., 2022). Zhuangzhuang Chu et al. designed a polypyrrole (Ppy) composite hybrid hydrogel. Incorporating Ppy into a hybrid hydrogel of PVA, PEG, and HA enhances macrophage phagosome activity and M1 activation by accumulating heat on the surface, accelerating bacterial clearance. Further, under NIR stimulation, macrophages within the hydrogel transition to the M2 phenotype, enhancing VEGF expression and collagen secretion, thereby promoting wound healing (Chu et al., 2025).

Combining Photothermal and ROS Responses: This combination achieves synergistic photothermal sterilization and ROS scavenging for improved wound healing outcomes. Yijia Xue et al. designed a NIR-responsive GelMA/CeO_2_/PDA hydrogel with antibacterial and antioxidant properties. PDA’s photothermal conversion under NIR irradiation promotes the smart release of the bioactive component CeO_2_ within the wound. This inhibits the interleukin (IL)-17 signaling pathway, eliminates local wound ROS, reduces apoptosis, and limits oxidative damage to proteins and cell membranes, facilitating the transition from the inflammatory to the proliferative phase, thus promoting cell proliferation and wound healing (Xue et al., 2025) (Figure 7e). Haoxin Cheng et al. designed an oral gel (MPCST) based on a TA-lipoic acid network, composed of PDA-coated molybdenum disulfide (MoS_2_) nanosheets (MoS_2_@PDA), the NO precursor sodium nitroprusside (SNP), and cerium oxide (CeO_2_). Under NIR irradiation, it rapidly heats up to release NO, promoting angiogenesis, scavenging ROS, and exerting antibacterial and anti-inflammatory effects to promote wound healing (Cheng et al., 2025). Xue Y et al. designed a NIR-responsive hydrogel composed of GelMA, CeO_2_, and PDA. The PDA component generates heat via photothermal conversion under NIR for antibacterial action. CeO_2_ scavenges excess ROS, alleviating oxidative stress and providing antioxidant effects. The hydrogel accelerates wound healing by providing an antibacterial and antioxidant microenvironment (Xue et al., 2025).

Combining Electrical Stimulation and Cytokine Responses: Sudan Liu et al. developed a PVA hydrogel incorporating Ti_3_C_2_T_x_ (MXene) and polyaniline (PANI) to enhance skin wound healing. Electrical stimulation (ES) promotes cell proliferation and migration. The hydrogel promotes healing by reducing pro-inflammatory cytokine expression, upregulating VEGF and α-SMA expression, and promoting angiogenesis and collagen deposition (Liu S. et al., 2022).

Combining pH and ROS Responses: Yutong Yang’s team prepared a cascade bacteria-responsive self-activating antibacterial composite hydrogel platform. It consists of an L-arginine-modified chitosan (CA) and phenylboronic acid-modified oxidized dextran (ODP) shell encapsulating a pH-responsive H_2_O_2_ self-supplying composite nanozyme (MSCO) and pH/enzyme-sensitive bacteria-responsive triblock micelles. Lactic acid produced by bacterial metabolism lowers the local pH, triggering the release of lactate oxidase from the pH-responsive hydrogel. This enzyme generates H_2_O_2_, which reacts with copper ions to produce ·OH for antibacterial action. Lactate oxidase also promotes NO release, enhancing antibacterial effects. Multiple inherent nanozymes in the hydrogel scavenge ROS, promoting wound healing (Yang et al., 2024).

Combining Light, Temperature, and pH Responses: This strategy uses multiple environmental triggers for precise control of drug release and photothermal effects, synergistically killing bacteria, clearing biofilms, and regulating the wound microenvironment. This efficiently inhibits infection and promotes tissue regeneration and wound healing. Chao Zhao et al. prepared a temperature-, pH-, and NIR-light multi-responsive hydrogel loaded with the drug doxorubicin and the photosensitizer ICG. It promotes wound healing by killing residual tumor cells, eliminating harmful bacteria, and disrupting bacterial biofilms (Zhao et al., 2021). Yuetong Zhang et al. constructed a novel biofilm microenvironment (BME)-responsive antibacterial platform based on tungsten (W)-polyoxometalate (POM) clusters. This platform features pH and glutathione dual-responsiveness, initiated by acidic BME-induced self-assembly and reductive BME-driven enhanced NIR absorption. It exerts photothermal antibacterial effects under NIR irradiation, collectively promoting wound healing (Zhang et al., 2020) (Figure 7f).

Combining Temperature, pH, and ROS Responses: This approach utilizes multiple microenvironmental triggers for intelligent drug release and material function switching, synergistically scavenging ROS, inhibiting bacteria, and promoting vascular and collagen regeneration. This efficiently remodels the wound microenvironment, accelerating tissue repair and healing. Xiao Han et al. prepared a double-network gel loaded with thermosensitive polymer-poly (N-isopropylacrylamide)/keratin (PNIPAAm/keratin DN), exhibiting temperature, pH, and ROS multiple responsiveness. It accelerates wound healing by promoting vascular regeneration, collagen deposition, and providing antibacterial and antioxidant effects (Han et al., 2021).

Combining pH Response, Photothermal Effect, and Pro-angiogenic Action: This strategy utilizes suitable microenvironmental triggers for intelligent drug release and photothermal conversion to synergistically kill bacteria, while continuously releasing active factors to promote angiogenesis. This efficiently clears pathogens, optimizes the regenerative microenvironment, and accelerates wound healing. Fei Wang et al. prepared a multifunctional sprayable hydrogel. It was formed by combining a nanoplatform of sequentially loaded Cu_2_Se (CS) and ciprofloxacin (CIP)-incorporated ZIF-8 (ZIF@CIP) copper-doped Ti MOF (CT) with a photocurable gel composed of chitosan methacrylate and gallic acid-grafted gelatin. The CS component has superior photothermal effects for preliminary antibacterial PTT. The strong pH sensitivity of the Ti MOF enables pH-responsive release of CIP for antibacterial action. Additionally, the hydrogel promotes angiogenesis and other functions to facilitate wound healing (Figure 7G) (Wang F. et al., 2025). Shuangli Zhu et al. prepared a metformin-loaded CuPDA-NPs composite hydrogel (Met@CuPDA-NPs/HG) via dynamic borate ester bonding between dopamine-modified gelatin (gel DA), copper-loaded PDA nanoparticles (CuPDA-NPs), and phenylboronic acid-modified HA. The primary structure involves borate ester bonds formed between phenylboronic acid groups and vicinal diols in glucose. These bonds are highly sensitive to pH and glucose levels and break easily under acidic conditions, giving the hydrogel pH-responsive properties. In a weakly acidic environment, more metformin is released to exert anti-inflammatory effects and promote healing. Furthermore, the hydrogel has photothermal antibacterial characteristics. The slow release of copper ions enables long-term antibacterial action and promotes angiogenesis, working together to promote wound healing (Zhu et al., 2023). In summary, integrating synergistic response mechanisms to endogenous microenvironmental signals and exogenous physical stimuli has become a core strategy for enhancing the clinical efficacy of intelligent oral dressings. The specificities of the oral environment, its moisture, complex microbiota, and frequent mechanical stress, make single-response modes inadequate for precise healing regulation. Combined response strategies, through multi-signal perception and feedback loops, significantly enhance the dressing’s adaptability to dynamic wound changes. This allows for the temporal activation of therapeutic functions and spatial targeting of delivery based on the healing stage and spatial heterogeneity.

### 4.4 Smart responsive self-healing materials

Oral wound repair has long been plagued by challenges such as poor adaptation to the moist and dynamic oral environment, recurrent inflammatory infections, and extended healing periods. Conventional dressings, lacking active regulatory functions and self-protective characteristics, often fall short in supporting the complex healing cascade. In contrast, smart responsive self-healing materials, relying on the reversible reorganization or cleavage of dynamic chemical bonds, can endure mechanical stresses induced by chewing and speech, thereby minimizing the risk of secondary injury associated with frequent dressing replacement. Moreover, by sensing specific microenvironmental cues at the wound site (e.g., pH, light, and mechanical forces), these materials can initiate functional transitions that enable precise drug release, modulation of mechanical properties, and enhanced repair efficiency. Shaojun Fang and colleagues developed a photo-responsive self-healing hydrogel via a two-step light-controlled cross-linking strategy. In the first stage, photoinitiation prompts rapid gelation, yielding a compliant matrix that adapts to the wound contours. Subsequent irradiation initiates secondary cross-linking, improving the mechanical integrity and tailoring the degradation profile to align with the oral mucosal healing process. This hydrogel facilitates the migration and proliferation of oral mucosal stem cells, attenuates inflammatory responses, and promotes angiogenesis and epithelial regeneration, collectively enhancing wound closure. The material exhibits no cytotoxicity and has demonstrated efficacious repair in both chronic oral ulcer and post-surgical wound models. However, the practical application of such hydrogels remains constrained by the sensitivity of the preparation process to annealing and ambient temperatures, which affects reproducibility. Further research is essential to optimize the fabrication protocol and improve the stability of the final product for clinical translation (Fang et al., 2025).

Chenglong Xue et al. constructed a multifunctional polysaccharide-based hydrogel (QCS/OHA-PEDOT-BBH-EGF) endowed with self-healing, pH-responsive, and antibacterial capabilities. The hydrogel network is formed through dynamic Schiff base linkages between amino groups in quaternized chitosan (QCS) and aldehyde groups in oxidized HA. Upon mechanical disruption, the reversible nature of the imine bonds enables rupture and reformation at the fractured interfaces, allowing autonomous self-healing without external intervention. Moreover, under NIR irradiation, the reorganization of Schiff base bonds is significantly accelerated, enabling the hydrogel to recover its structural integrity even after repeated sectioning. This photo-enhanced reparability effectively extends the functional lifespan of the dressing, highlighting its potential for sustained therapeutic application (Xue et al., 2022).

Chenggui Wang et al. designed an injectable, self-healing, antibacterial, and long-term pH-responsive peptide-based FHE hydrogel (F127/OHA-EPL) for enhanced chronic wound repair via the incorporation of adipose-derived mesenchymal stem cell exosomes (AMSCs-exo). The hydrogel network is constructed through dynamic Schiff base bonds formed between aldehyde groups in oxidized HA and amino groups in ε-poly-L-lysine (EPL). Under mechanical stress, the reversible Schiff base linkages dissociate temporarily; when fractured surfaces come into contact, the exposed functional groups readily reestablish covalent connections, enabling rapid structural recovery and autonomic self-healing. During the inflammatory phase, as the local wound pH drops to 5.5–6.0, the acidic microenvironment triggers protonation and cleavage of the Schiff base bonds, leading to network loosening and controlled release of AMSCs-exo. Under physiological pH (∼7.4), release is sustained at a slower rate, achieving a smart inflammatory-phase burst release and healing-phase sustained release profile that prolongs exosome bioavailability. This pH-modulated release behavior significantly enhances re-epithelialization, promotes organized collagen deposition, and works synergistically with the intrinsic self-healing property to improve healing outcomes in chronic wounds (Wang et al., 2019). However, both the self-healing hydrogels exhibit limited adaptability to the intraoral wound environment. Future efforts should focus on enhancing material compliance under moist and dynamic oral conditions, as successful environmental adaptation is essential for realizing their full potential as smart antibacterial wound dressings.

### 4.5 *In vitro* and *in vivo* models

The *in vitro* evaluation is fundamental for the initial assessment of smart stimuli-responsive materials. Conventional two-dimensional (2D) cell cultures of relevant cell types, such as oral keratinocytes and fibroblasts, provide a high-throughput platform for preliminary analyses of cytocompatibility, cell adhesion, migration, and proliferation. However, to accurately mimic the *in vivo* environment, 3D culture systems are increasingly employed. These 3D models, including cell spheroids or scaffolds seeded with multiple cell types, offer superior insights into cell-material interactions and the assessment of pro-angiogenic or anti-fibrotic properties. For a more rigorous evaluation that captures the complexity of infected oral wounds, sophisticated models such as biofilm-infected coculture systems are critical. These involve pre-establishing bacterial biofilms on the material surface or within a 3D matrix, allowing for the simultaneous assessment of the material’s antibacterial efficacy and its ability to support host cell function under challenge. Furthermore, the emergence of oral mucosa-equivalent organoids represents a cutting-edge tool. These patient-derived mini-tissues recapitulate the stratified epithelium and can be used to validate key therapeutic functions, such as the pH-triggered release of antimicrobials or growth factors in a pathologically relevant microenvironment, thereby providing highly predictive data before proceeding to *in vivo* studies.

The translation of smart hydrogels from *in vitro* validation to clinical application necessitates robust *in vivo* testing. Rodent models, particularly in rats or mice, are the most widely used due to their cost-effectiveness, genetic manipulability, and well-established protocols for creating oral mucosal wounds. These models are indispensable for proof-of-concept studies, initial biocompatibility screening, and understanding fundamental healing mechanisms. However, they have inherent limitations, including the relatively static oral environment and anatomical differences from humans. To address these shortcomings, larger animal models such as porcine or canine are employed. The oral anatomy, tissue biomechanics, and healing processes in these species more closely resemble those of humans. Crucially, their larger oral cavity and dynamic environment, subject to constant salivary flow, masticatory forces, and microbial challenge, provide a far more rigorous and clinically relevant setting to evaluate a material’s critical performance metrics. This includes its ability to maintain adhesion and structural integrity under mechanical stress, its responsiveness to dynamic pH fluctuations, and its long-term therapeutic efficacy in promoting functional tissue regeneration with minimal scarring. Therefore, a tiered approach utilizing rodent models for initial screening followed by validation in a physiologically relevant large animal model is considered the gold standard for preclinical assessment of smart dressings for oral wound repair.

## 5 Challenges and future perspectives

The clinical translation of smart dressings for oral wound repair faces several interconnected challenges. First, inherent material limitations persist, including the fundamental trade-off between self-healing efficiency and mechanical strength in dynamic hydrogel networks, along with the slow response kinetics of stimuli-sensitive mechanisms and inadequate adhesive performance in the wet, mobile oral environment. Second, significant translational hurdles exist, such as scalable and reproducible manufacturing, the development of sterilization methods compatible with dynamic bonds and bioactive cargo, and navigating complex regulatory pathways for combination products. Third, while short-term cytocompatibility is frequently reported, comprehensive data on long-term biosafety, including chronic immune responses to degradation products, the biological fate of embedded nanoparticles, and the long-term impact of material residues, are critically lacking. Finally, the performance of these materials under the complex oral milieu, involving enzymatic activity, microbiota, and mechanical stresses, remains difficult to predict using standard laboratory models, necessitating more biomimetic evaluation platforms.

The future trajectory of smart responsive materials for oral wound repair is poised to be revolutionized by the convergence of advanced manufacturing and digital health technologies, particularly through the integration of 3D bioprinting and artificial intelligence (AI). This synergy promises to shift the paradigm from a one-size-fits-all approach to truly personalized and adaptive wound management.

In the realm of advanced manufacturing, 3D bioprinting transcends simple scaffold fabrication. It enables the creation of constructs with patient-specific geometry derived from intraoral scanning, tailored hierarchical porosity to guide tissue ingrowth, and most importantly, spatially graded bioactivity. By formulating bioinks from the smart hydrogels discussed previously, we can engineer architectures that not only conform perfectly to the complex wound topography but also execute sophisticated therapeutic programs. For instance, a single printed construct could be designed with a core layer laden with antimicrobial peptides that release in response to bacterial enzymes, while a surface layer releases growth factors like EGF or VEGF in a pH-dependent manner during the inflammatory phase. The emerging concept of 4D bioprinting, where printed structures evolve over time in response to stimuli, could further allow the scaffold to dynamically change its morphology or stiffness to actively participate in the healing process. In parallel, AI-driven approaches are set to unlock a new level of personalized therapy. The future management of oral wounds will likely involve the continuous collection of multi-modal patient data. This includes static data such as genomic predisposition to inflammation or diabetic status, and dynamic, real-time data from wearable or even integratable biosensors monitoring wound pH, temperature, moisture, and specific biomarkers in saliva. Machine learning models, trained on vast datasets of wound outcomes, can analyze this information to predict individual healing trajectories, identify early signs of infection or healing stagnation, and dynamically optimize treatment parameters. Such a closed-loop system could, for example, autonomously adjust the dosage of a drug released from the smart dressing or trigger an external stimulus (e.g., a specific wavelength of light) to modulate the material’s behavior. AI can also accelerate the discovery of next-generation smart materials by screening vast chemical libraries for optimal responsive properties and biocompatibility.

However, the path forward necessitates addressing key challenges. For 3D bioprinting, these include improving the printability and structural integrity of smart hydrogel bioinks under physiological conditions. For AI, the challenges encompass the need for large, high-quality, and standardized clinical datasets, and ensuring the robustness and clinical interpretability of the algorithms. Ultimately, the synergistic combination of 3D bioprinting for bespoke structural and biochemical design, and AI for intelligent, data-driven regulation of the treatment process, is anticipated to usher in a new era of precision wound care. This integrated approach will fundamentally improve the management of complex oral wounds by creating systems that are not just smart, but truly intelligent and adaptive.

## 6 Conclusion

The oral cavity presents a complex wound environment, characterized by its natural microbial state, high ROS levels, low pH, abnormal MMP activity, high humidity, and frequent movement of surrounding tissues. These factors easily lead to delayed healing and increased risk of infection, posing significant challenges for clinical management. As a crucial tool in oral wound care, current wound dressings have notable limitations: traditional medical hydrogels struggle to adapt to the unique oral environment, and their anti-infective efficacy and application reliability remain debatable. In recent years, smart responsive wound dressings have demonstrated considerable advantages. These dressings can respond to local wound microenvironment changes (such as pH, ROS, enzymes or temperature) to achieve controlled and ordered drug release, actively improve the healing environment, and some even offer analgesic effects. Such dressings show excellent compatibility in the moist, microbially complex, and mechanically active oral environment. Their “on-demand response” property not only overcomes the limitations of traditional dressings, such as poor adhesion, drug burst release, and short antibacterial duration, but also simulates the dynamic physiological processes of wound healing, optimizing the microenvironment for hemostasis, anti-infection, anti-inflammation, and tissue regeneration. Furthermore, they may reduce overall treatment costs in terms of treatment duration and frequency of changes, indicating broad potential for clinical application. However, these dressings are still primarily in the preclinical research stage and are not yet widely used. Their main shortcomings include: as drug carriers, their release behavior is susceptible to interference from various factors in the oral environment, such as pH and enzyme activity—particularly for multi-stimuli responsive dressings, which require systematic evaluation of the impact of various variables on drug release kinetics. Simultaneously, their formability, drug loading stability, and physicochemical stability during storage and transportation need further improvement. On the other hand, wound healing itself is a dynamic process with continuously fluctuating microenvironment parameters (e.g., pH, ROS). Coupled with inter-patient variability and differences across age groups, these factors can affect the consistency of the dressing’s response and its therapeutic efficacy.
